# Weather-Related Disaster in a Diverse Cohort of Aging Adults

**DOI:** 10.1093/geroni/igab046.2876

**Published:** 2021-12-17

**Authors:** Yvonne Michael, Lauren Clay, Kevin Smilely, Rennie Joshi, Jana Hirsch

**Affiliations:** 1 Drexel University, PHILADELPHIA, Pennsylvania, United States; 2 D'Youville College, Buffalo, New York, United States; 3 Louisiana State University, Baton Rouge, Louisiana, United States; 4 Drexel University, Philadelphia, Pennsylvania, United States

## Abstract

As climate change contributes to increasing frequency and intensity of weather-related disasters, it is critical to define characteristics that increase risk of poor health outcomes during and after events. Given the aging of the United States (US) population and over-representation of older adults in disaster-prone areas, disaster-related impacts on older adults present a growing public health challenge. We linked data from the REGARDS study, a cohort of 30,107 Black and White adults (mean age 65 years at baseline, 2003-2007), with community data from the National Establishment Time Series database and longitudinal weather-related disaster data from the Spatial Hazard Events and Losses Database for the US. We calculated disaster exposure for each year for the county in which the respondents lived from 2003 – 2015: 84% of county-years showed at least some impacts, including 16% of counties experiencing medium impacts ($10- $50 property damage per capita or 2 fatalities) and 12% severe (greater than $50 per capital or 3 fatalities); this mirrors that of the continental US (77% some impact, 15% medium, 13% severe). REGARDS participants exposed to moderate or severe disasters were more likely to be Black and low socioeconomic status compared to those who were not exposed. For community characteristics, higher disaster exposure was associated with a greater density of resources including ambulatory care, food stores, social services, and destinations for daily living. Our approach showcases how disaster preparedness systems need better data about specific individual-and community-level factors that increase risk among older adults to better serve communities.

